# Chest trauma revealed an ostium secundum atrial septal defect in adulthood

**DOI:** 10.11604/pamj.2014.17.29.3295

**Published:** 2014-01-17

**Authors:** Zine el abidine Benali, Hatim Abdedaim

**Affiliations:** 1 Department of Anesthesiology, CHP Eddarak, Berkane, Morocco; 2 Department of Anesthesiology, Military Hospital Mohammed V, University Mohammed V Souissi, Rabat, Morocco

**Keywords:** Chest trauma, ostium secundum, atrial septal defect

## Image in medicine

Atrial septal defect (ASD) is characterized by a defect in the interatrial septum allowing pulmonary venous return from the left atrium to pass directly to the right atrium. ASD account for 10% of all congenital heart disease, and as much as 30% of congenital heart disease presenting in adulthood. There are four types of ASD that can be recognized as the seat of the port: ostium secundum, the most common, they are located in the area of the oval fossa, and are usually well focused, to differentiate from a patent foramen ovale as in our patient, isolated secundum atrial septal defects account for approximately 7% of congenital cardiac defects; sinus venosus, high location, near to the anastomosis of the pulmonary veins; ostium primum, low, can be integrated into a more complete atrioventricular canal; coronary sinus, a very rare form, this type is a fenestration or a total absence of the roof of the coronary sinus, into the left atrium. In general, elective closure is advised for all ASD with evidence of right ventricular overload or with a clinically significant shunt (pulmonary flow [Qp] to systemic flow [Qs] ratio >1.5). Closure of an ASD is not recommended in patients with a clinically insignificant shunt (Qp-Qs ratio 0.7 or below) and in those who have severe pulmonary arterial hypertension or irreversible pulmonary vascular occlusive disease who have a reversed shunt: right to left with hypoxemia. We report the case of a men aged 35 years, admitted to the ICU for a left pneumo-hemothorax with rib fracture resulting from an accident of the highway, drained emergency. Auscultation of the heart sounds: a systolic ejection murmur, respiratory variations in the splitting of the second heart sound; in the ECG: presence of incomplete right bundle branch block with atrial flutter. The echocardiography at the bedside with multiple effects showed dilatation of the right ventricle, and confirming the atrial septal defect type ostium secundum with left-right shunt noting that the subcostal window is a better impact on the diagnostic to ask for two reasons: ultrasound are perpendicular, Doppler is strictly aligned. Hypertension of pulmonary artery measured at 45 mmHg. The patient is referred to the cardiovascular consultation after its release for therapeutic cardiology discussion.

**Figure 1:**
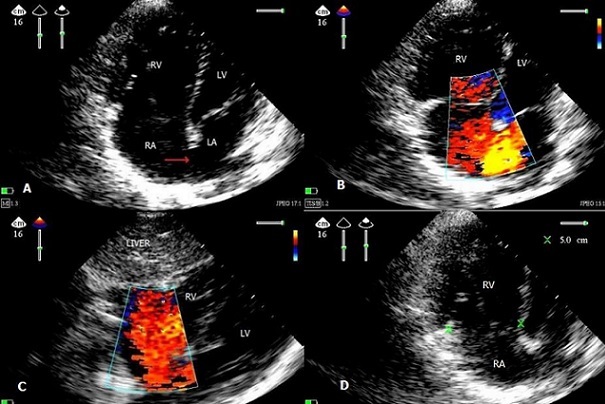
Ultrasound images A) apical four cavity 2d echocardiography image showing dilatation of right ventricular with rv / lv higher than 0.6 in diameter and surface, atrial septal defect with 3 cm type ostium secundum; B) apical four cavity with color doppler showing a left - right shunt; C) the subcostal window with color doppler showing the atrial septal defect with shunt; D) apical four cavity centred on the right ventricle showing the expansion of the ring tricuspid to 5 cm. (LA: left atrium, LV: left ventricle, RV: right ventricle, RA: right atrium, Red arrow: atrial septal defect)

